# Radiomics Feature Analysis of Cartilage and Subchondral Bone in Differentiating Knees Predisposed to Posttraumatic Osteoarthritis after Anterior Cruciate Ligament Reconstruction from Healthy Knees

**DOI:** 10.1155/2021/4351499

**Published:** 2021-09-12

**Authors:** Yuxue Xie, Yibo Dan, Hongyue Tao, Chenglong Wang, Chengxiu Zhang, Yida Wang, Jiayu Yang, Guang Yang, Shuang Chen

**Affiliations:** ^1^Department of Radiology & Institute of Medical Functional and Molecular Imaging, Huashan Hospital, Fudan University, 12 Middle Wulumuqizhong Road, Shanghai 200040, China; ^2^Shanghai Key Laboratory of Magnetic Resonance, East China Normal University, 3663 N. Zhongshan Rd, Shanghai 200062, China; ^3^National Clinical Research Center for Aging and Medicine, Huashan Hospital, Fudan University, Shanghai 200040, China

## Abstract

**Objectives:**

To introduce a new implementation of radiomics analysis for cartilage and subchondral bone of the knee and to compare the performance of the proposed models to classic T2 relaxation time in distinguishing knees predisposed to posttraumatic osteoarthritis (PTOA) after anterior cruciate ligament reconstruction (ACLR) and healthy controls.

**Methods:**

114 patients following ACLR after at least 2 years and 43 healthy controls were reviewed and allocated to training (*n* = 110) and testing (*n* = 47) cohorts. Radiomics models are built for cartilage and subchondral bone regions of different compartments: lateral femur (LF), lateral tibia (LT), medial femur (MF), and medial tibia (MT) and combined models of four compartments on T2 mapping images. The model performance of discrimination between patients and controls was illustrated with the receiver operating characteristic curve and compared with a classic T2 value-based model.

**Results:**

The T2 value model of cartilage yielded moderate predictive performance in discerning patients and controls, with an AUC of 0.731 (95% confidence interval, 0.556–0.875) in the testing cohort, while the radiomics signature of cartilage and subchondral bone of different compartments demonstrated excellent performance, with AUCs of 0.864–0.979. Furthermore, the combined model reported an even better performance, with AUCs of 0.977 (95% confidence interval, 0.919–1.000) for the cartilage and 0.934 (95% confidence interval, 0.865–0.994) for the subchondral bone in the testing cohort.

**Conclusion:**

The radiomics features of the cartilage and subchondral bone may be able to provide powerful tools with more sensitive detection than T2 values in differentiating knees at risk for PTOA after ACLR from healthy knees.

## 1. Introduction

Posttraumatic osteoarthritis (PTOA), a subtype of OA, develops after intraarticular injury in a disproportionately young population. Identifying the earliest stages of onset of PTOA provides the greatest opportunity for prevention and treatment of this disease. Although articular cartilage degeneration has been regarded as the onset of PTOA for a long time [1], a few animal models have demonstrated that subchondral bone changes precede articular cartilage changes in the process of PTOA after anterior cruciate ligament reconstruction (ACLR) [2–5].

Quantitative MRI parameters, such as T2 relaxation time, have been well established to be able to assess cartilage degeneration and therefore were proved to be an early biomarker of PTOA in the knee after ACLR [6, 7]. By contrast, no robust tool is available to assess the subchondral bone changes in early stages of the disease, in part because of the lack of appropriate imaging techniques that can properly detect the subchondral bone characteristic. Radiomics, which extracts hundreds or thousands of features from medical images and builds models upon them, has shown promise in automating image analysis and discovering subtle features associated with disease [8]. This technique was utilized in the cartilage and subchondral bone analysis in knee OA recently [9, 10]. For example, Birch et al. found significant differences between the tibial fractal signatures in the medial and lateral compartments following ACL rupture and those of uninjured controls [9].

The aim of this study is at constructing and testing a radiomics model in cartilage and subchondral bone and at comparing the performance of the proposed models to classic T2 relaxation time in distinguishing patients predisposed to PTOA after ACLR and healthy controls. We expected that cartilage and subchondral bone architecture based on radiomics analysis can reveal more robust characteristics of the knees at risk for PTOA than T2 relaxation time of cartilage degeneration in patients after ACLR.

## 2. Materials and Methods

### 2.1. Study Subjects

This retrospective study was approved by our institutional review board, and the requirement for the written informed consent was obtained from all participants (patients and controls). A total of consecutive 114 patients after ACLR treated from 2013 to 2017 in a single center were enrolled in this study. The inclusion criteria were (1) clear evidence of unilateral ACL rupture as shown by MRI or clinical examination and confirmed by arthroscopy, (2) no prior injury both in the ipsilateral and in the contralateral knees, (3) age between 18 and 40 at the time of rupture, and (4) body mass index (BMI) below 25 kg/m^2^. The exclusion criteria comprised knee OA, inflammatory arthritis, and cartilage damage confirmed by arthroscopy, combined with other injuries such as posterior cruciate ligament tear, medial or lateral collateral ligament injury, and serious meniscus injury. A group of healthy controls (*n* = 43) without any injury or disease in the knee was recruited, matching the patients with respect to sex, age, and BMI.

### 2.2. MRI Scanning, Cartilage Segmentation, and Subchondral Bone Segmentation

The patients underwent MRI examination postoperatively at least 2 year after ACLR. The controls underwent MRI examination on the nondominant knee. MRI scans were performed on a 3T MRI unit (Verio, Siemens, Germany) with an 8-channel phase array knee coil. Knee cartilage was scanned in the sagittal plane using T2 mapping (TR/TE = 1523/13.8, 27.6, 41.4, 55.2, and 69.0 ms, FOV read = 160 mm, and slice thickness = 3 mm).

**Figure 1:**
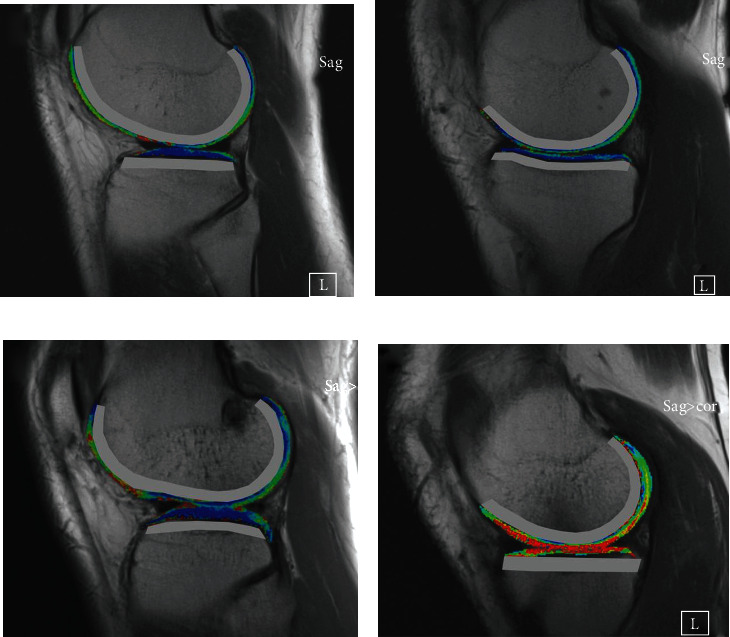
Cartilage and subchondral bone range of interest (ROI) of different compartments. (a) Lateral femur and tibia ROI of a healthy control; (b) medial femur and tibia ROI of the control; (c) lateral femur and tibia ROI of an ACLR patient; (d) medial femur and tibia ROI of the patient. The ROI of cartilage was segmented by a radiologist, representing T2 values (blue for 0 ms and red for 100 ms). The ROI of subchondral bone, which is a 5 mm-wide band-like structure below the subchondral bone plate covered by cartilage, was calculated according to the cartilage ROI and illustrated with a grey border.

The cartilage regions of interest (ROI) were manually contoured on T2 mapping images using ITK-SNAP (v. 3.6.0; http://www.itksnap. org; open-source software). The femoral cartilage and the tibial cartilage were divided into four compartments (medial femur (MF), medial tibia (MT), lateral femur (LF), and lateral tibia (LT)). The calculated T2 mean values (Figure S1, (supplemental)) of four cartilage regions were used as features to establish the LR classification model directly. The ROI for the subchondral bone of different compartments was a 5 mm-wide band-like structure below the subchondral bone plate covered by cartilage ROI [11] (Figure 1). The subchondral bone ROIs were calculated geometrically from the corresponding cartilage ROIs segmented by the radiologist. Radiomics models are built for cartilage and subchondral bone regions of different compartments: lateral femur (LF), lateral tibia (LT), medial femur (MF), and medial tibia (MT) and combined models of four compartments on T2 mapping images.

### 2.3. Radiomics Feature Extraction and Model Construction

Two radiologists (radiologist 1 with 5 years and radiologist 2 with 10 years of experience) performed the same delineation of the ROI. Radiologist 1 delineated the ROI twice at different times and radiologist 2 carried out the delineation once. The radiomics features were calculated after each delineation and intra- and interobserver reproducibility determined for each feature. Features with low reproducibility (intra- or interobserver intraclass coefficient (ICC) below 0.75) were excluded [12]. Radiomics features of the cartilage and the subchondral bone were extracted from the original image (normalized to the range of [0, 100]), wavelet transform, and Laplacian of Gaussian (LoG) transform. Classes of features extracted included first-order and texture features. We used the Synthetic Minority Oversampling Technique (SMOTE) to balance positive and negative samples. Then, to remove redundant features, we used the Pearson correlation coefficient (PCC) analysis. Before building of the final model, recursive feature elimination (RFE) was used to select features. We used logistic regression as the classifier. Logistic regression is a linear classifier that combines all the features. A hyperplane was searched in the high dimension to separate the samples. To determine the number of features to be kept in the model, we used a 5-fold crossvalidation over the training data and the validation AUC was plotted against the number of features. The number of features corresponding to the best validation AUC was used for the model. After that, features used in the model that yielded the best validation AUC were combined to construct the combined radiomics models of cartilage and subchondral bone. Finally, all the models of interest were assessed with the test dataset for their classification performance. All of the above processes were implemented using an open-source free software, FeatureExplorer (ver. 0.3.6), which used PyRadiomics (ver. 3.0, http://pyradiomics.readthedocs.io/en/latest/index.html) and scikit-learn (ver. 0.22.2) in the back for feature extraction and model building, respectively [13].

### 2.4. Statistical Analysis

The statistical analysis was carried out using SciPy, in Python (v.3.6.5; http://www.python.org/). *P* values < 0.05 were considered to indicate a significant difference. Categorical variables were reported as numbers and percentages, and continuous variables were reported as mean ± standard deviation. Categorical variables were compared by the *χ*^2^ test or the Fisher's exact test, and continuous variables were compared by the independent-sample *t*-test or the Mann–Whitney *U*-test.

## 3. Results

### 3.1. Study Subject Characteristics

**Figure 2:**
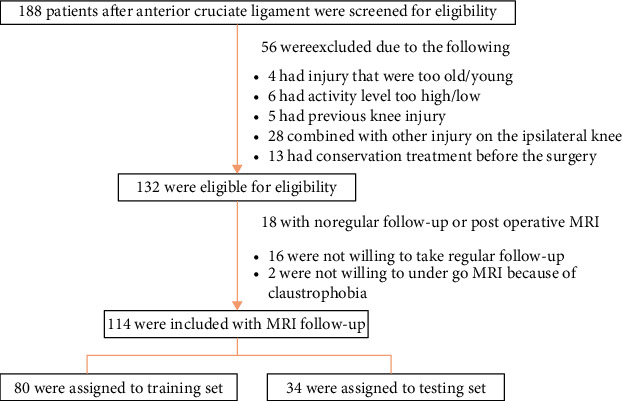
Flowchart showing the patient selection.

The flow of the patient selection process is displayed in Figure 2. The patients and the controls were randomly divided into a training set (110 cases, patients/controls = 80/30) and a testing set (47 cases, patients/controls = 34/13) (Table 1). The demographic characteristics for the training group and testing group are summarized in Table 1. No significant differences were displayed between the patient group and the control group regarding age, gender, and body mass index (*P* > 0.05). Neither between the training and testing cohorts (*P* > 0.05).

### 3.2. Radiomics Feature Extraction and Selection

The features extracted included first-order features and texture features. A total of 1116 features from cartilage and subchondral bone of different compartments were extracted from T2 maps. We used PCC for feature dimensionality reduction and RFE for feature selection. Nineteen features were selected for the combined cartilage radiomics model (Figure S2, (supplemental)) and 13 features for the combined subchondral bone radiomics model (Figure S3, (supplemental)). The features and their corresponding weights were reported in Table S1 and S2 (supplemental).

### 3.3. Performance of the T2 Value Model and the Combined Radiomics Models

**Figure 3:**
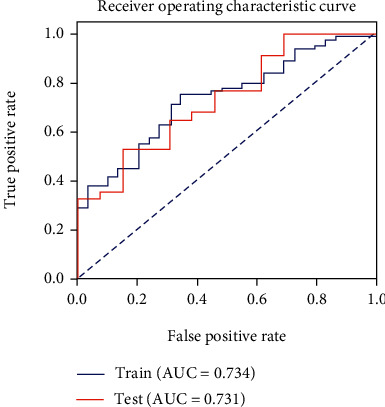
The receiver operating characteristic curve of the cartilage T2 value model in both the training and testing cohorts. The T2 value reached an AUC of 0.734 in the training cohort and an AUC of 0.731 in the testing cohort.

**Figure 4:**
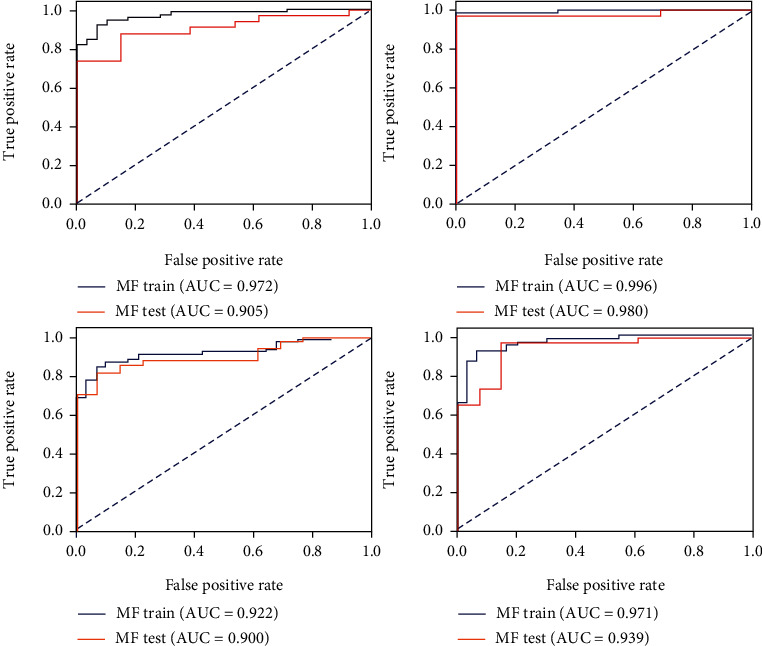
The receiver operating characteristic curves of the cartilage radiomics model in both the training and testing cohorts of different compartments. MF: medial femur; LF: lateral femur; MT: medial tibia; LT: lateral tibia.

**Figure 5:**
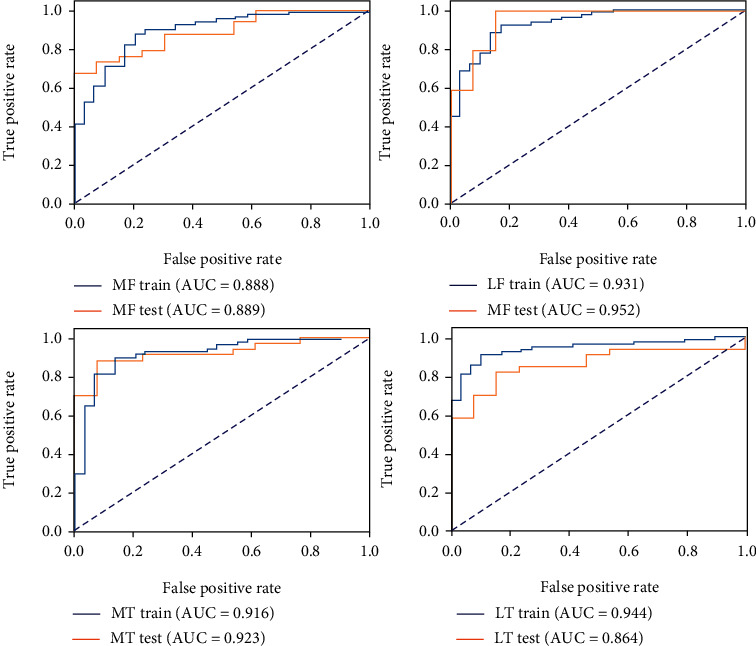
The receiver operating characteristic curves of the subchondral bone radiomics models in both the training and testing cohorts of different compartments. MF: medial femur; LF: lateral femur; MT: medial tibia; LT: lateral tibia.

**Figure 6:**
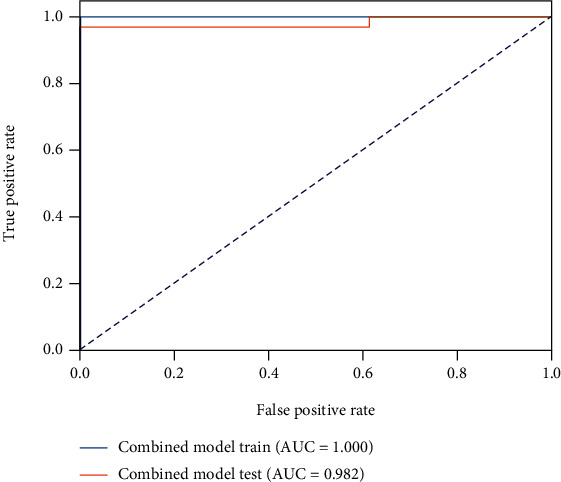
The receiver operating characteristic curve of the combined cartilage radiomics model in both the training and testing cohorts.

**Figure 7:**
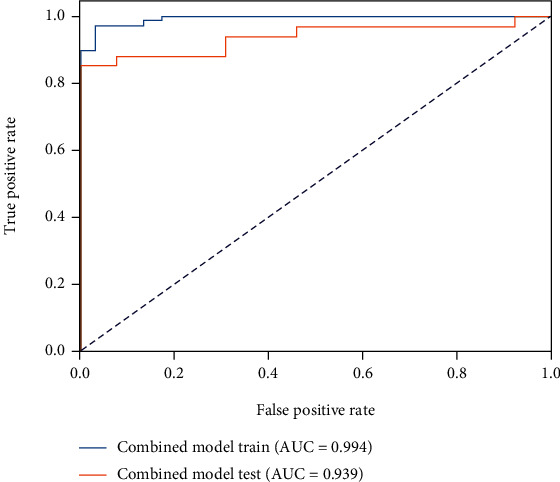
The receiver operating characteristic curve of the combined subchondral bone radiomics model in both the training and testing cohorts.

The T2 value model demonstrated moderate capability to discern ACLR patients and healthy controls, with an AUC of 0.731 in the testing cohort (Figure 3), while the cartilage radiomics model and the subchondral bone radiomics models in different compartments yielded excellent predictive power (Figures 4 and 5). Furthermore, the combined cartilage model and the subchondral bone model yielded an even better performance with AUCs of 0.982 and 0.939, respectively (Figures 6 and 7). Detailed information about the prediction performance of the T2 value model and radiomics models of cartilage as well as subchondral bone is shown in Tables 2–4.

## 4. Discussion

In this study, radiomic analysis was used to probe the subsurface information of cartilage as well as subchondral bone from MRI T2 mapping in patients at increased risk of developing PTOA. The performance of the proposed radiomics models was evaluated against typical T2 value analysis. With higher AUCs and better accuracy compared to T2 values of cartilage in classifying patients after ACLR and healthy controls, radiomics features of the subchondral bone and cartilage may be able to provide a powerful tool with more sensitive detection than T2 values in discerning knees at risk for PTOA after ACLR and healthy knees. This study suggested that radiomics analysis seems to be able to provide additional values of T2 mapping for charactering early PTOA in patients after ACLR, apart from typical T2 values.

Unlike primary OA, PTOA represents a cause of functional disability in a disproportionately young population because primary injuries are more likely to be sustained by younger individuals [14]. Moreover, PTOA commonly has a known “starting point,” which means that interventions could theoretically be initiated at an early stage to prevent the progression of the disease [15]. Therefore, there is a more compelling need to improve diagnostic techniques in order to detect PTOA at an early stage. T2 mapping has made the greatest strides in the evaluation of cartilage degeneration, which is a well-established early indicator of PTOA in patients after ACLR [16–18]. However, mean T2 value analysis is somewhat subject to many common limitations, such as dependency of loading [19], sequence type [20], or parameter selection [21]. Moreover, the T2 values vary between sites and vendors [22, 23]. On the other hand, many studies suggest radiomics as an interesting prospect to be studied regarding quantitative MRI images without these shortcomings aforementioned [24]. The superior performance of radiomics features to T2 values of cartilage in discerning ACLR patients and controls indicated that radiomics features showed higher sensitivity in assessing the degenerative changes of early-stage PTOA in knee cartilage after ACLR compared to the T2 values. To our knowledge, the previous work of Peuna et al. is the only other work on radiomics analysis of quantitative MRI T2 maps in a knee OA cohort [10]. They demonstrated texture analysis methods based on GLCM of articular cartilage and reported that texture analysis provided a powerful tool for assessment of knee OA with more sensitive detection of cartilage degeneration compared to the mean T2 value. Although their application of primary OA differed from ours, they ended up with quite similar findings regarding better performance of radiomics features than T2 values, which might indicate that our findings are not specific only to our method and data but are feasibly transferable to further studies.

Although articular cartilage degeneration has been regarded as a main cornerstone of PTOA, the disease is now increasingly recognized as being related to subchondral bone [25]. Therefore, we further explored the radiomics features of chondral bone to provide additional information regarding subchondral changes associated with PTOA in ACLR knees. To develop the radiomics model of subchondral bone, 13 most relevant radiomics features were selected from 1116 radiomics features by Pearson correlation coefficient and RFE, including grayscale and texture features of wavelet from T2 mapping images. As a result, the radiomics model yielded excellent predictive performance in identifying ACLR patients predisposed to PTOA. This result supported the view that the subchondral bone characteristic is consistent with changes associated with early PTOA. This result is similar with one previous study focusing on PTOA progression after ACLR [26]. In their study, the bone microarchitecture in ACLR knees of injured female participants was assessed by high-resolution peripheral quantitative CT (HR-pQCT) and compared directly to their uninjured contralateral knees, as well as to a healthy age-matched female control sample. Lower trabecular bone mineral density was found in the ACLR knees, indicating that subchondral bone features are consistent with changes associated with PTOA progression. Besides, in the testing cohort, the radiomics model of subchondral bone produced remarkable a higher AUC value and prediction accuracy than the T2 value model, which indicated that radiomics features of the subchondral bone provided a powerful tool with more sensitive detection than cartilage degeneration in characterizing early knee PTOA. It demonstrated that quantitative MRI T2 mapping can be used as an in vivo tool for the simultaneous imaging of cartilage and subchondral bone focusing on the early PTOA change in patients after ACLR. Thus, MRI T2 mapping can play a more important role in the early diagnosis of PTOA than it used to do.

There are several potential limitations for this study. First, it is a cross-sectional study based on a relatively small number of subjects, so a potential selection bias or unknown confounding factors may be present. This pilot study explored the possibility of radiomic analysis using T2 mapping to characterize PTOA. In the future, a longitudinal study should further be performed to explore the change in subchondral bone features as well as in cartilage in longer follow-ups. The small sample size also prevented us from doing a subanalysis with other injuries such as meniscal tears which may contribute to outcomes. Secondly, the subchondral bone features were not pathologically confirmed, due to ethical problems always faced by the study of the human joint, instead of tumors. Although it is tempting to make assumptions about the importance of subchondral bone changes in PTOA, any such conclusions should be made with caution. A longitudinal study would be needed to follow changes in the joint to determine the function of subchondral bone in the pathogenesis of PTOA after ACLR.

In conclusion, radiomics features of the cartilage and subchondral bone may be able to provide powerful tools with more sensitive detection than typical T2 values in differentiating knees at risk for PTOA after ACLR from healthy knees.

## Figures and Tables

**Table 1:** Demographic data of participants in the ACLR group and control group.

	Training cohort (*n* = 110)			Testing cohort (*n* = 47)		
	ACLR group	Control group	*P* value	ACLR group	Control group	*P* value
Number, *n*	80	30	/	34	13	/
Age, mean ± SD, *y*	26.875 ± 4.132	24.312 ± 3.225	0.553	24.312 ± 3.225	26.329 ± 2.789	0.602
Sex, male/female, *n*	77/3	26/4	0.162	31/3	12/1	1.000
BMI, mean ± SD, kg/m^2^	23.845 ± 1.990	23.712 ± 2.374	0.654	23.992 ± 1.548	23.932 ± 2.358	0.798
Left/right, *n*	49/31	20/10	0.763	19/15	9/4	0.619
Injury duration (mo)	4.125 ± 2.250	/	/	4.785 ± 2.375	/	/

ACLR: anterior cruciate ligament reconstruction; BMI: body mass index; SD: standard deviation.

**Table 2:** The performance of the T2 value model of cartilage.

Statistics	Value
Accuracy	0.617
AUC	0.731
AUC 95% CIs	0.556–0.875
NPV	0.407
PPV	0.900
Sensitivity	0.529
Specificity	0.846

AUC: area under the receiver operating characteristic curve; NPV: net present value; PPV: positive predictive value.

**Table 3:** The performance of the cartilage radiomics models of different compartments and combined cartilage.

Statistics	MF	LF	MT	LT	Combined cartilage
Accuracy	0.809	0.979	0.851	0.936	0.979
AUC	0.905	0.980	0.900	0.939	0.982
AUC 95% CIs	0.803–0.979	0.925–1.000	0.803–0.976	0.854–1.000	0.919–1.000
NPV	0.591	0.928	0.667	0.917	0.929
PPV	1.000	1.000	0.966	0.943	1.000
Sensitivity	0.735	0.971	0.824	0.971	0.971
Specificity	1.000	1.000	0.923	0.846	1.000

MF: medial femur; LF: lateral femur; MT: medial tibia; LT: lateral tibia; AUC: area under the receiver operating characteristic curve; NPV: net present value; PPV; positive predictive value.

**Table 4:** The performance of subchondral bone radiomics models in different compartments and combined subchondral bone.

Statistics	LF	LT	MF	MT	Combined subchondral bone
Accuracy	0.766	0.957	0.894	0.829	0.894
AUC	0.889	0.952	0.923	0.864	0.939
AUC 95% CIs	0.791–0.971	0.866–1.000	0.833–0.988	0.742–0.961	0.865–0.994
NPV	0.541	1.000	0.750	0.647	0.722
PPV	1.000	0.944	0.967	0.933	1.000
Sensitivity	0.676	1.000	0.882	0.824	0.853
Specificity	1.000	0.846	0.923	0.846	1.000

MF: medial femur; LF: lateral femur; MT: medial tibia; LT: lateral tibia; AUC: area under the receiver operating characteristic curve; NPV: net present value; PPV: positive predictive value.

## Data Availability

The [T2mapping] data used to support the findings of this study are available from the corresponding author upon request.

## Supplementary Materials

Supplementary Materials Figure S1: the distribution of cartilage T2 values of different compartments of the knee. MF: medial femur; LF: lateral femur; MT: medial tibia; LT: lateral tibia. Figure S2: (a) combined cartilage radiomics model on feature select, 19 features which correspond to the optimal validation AUC were selected; (b) radiomics score plot on test data set. Figure S3: (a) combined subchondral radiomics model on feature selection, 13 features which correspond to the optimal validation AUC were selected; (b) radiomics score plot on test data set. Table S1: features and weights of the combined cartilage radiomics model. Table S2: features and their corresponding weights in the combined subchondral radiomics model.
