# Supplementary material for: Radiomics Feature Analysis of Cartilage and Subchondral Bone in Differentiating Knees Predisposed to Posttraumatic Osteoarthritis after Anterior Cruciate Ligament Reconstruction from Healthy Knees
Source: Biomed Res Int. 2021 Sep 12;2021:4351499. doi: 10.1155/2021/4351499 (PMC8452399; doi:10.1155/2021/4351499)
Supplement: Supplementary Materials — Figure S1: the distribution of cartilage T2 values of different compartments of the knee. MF: medial femur; LF: lateral femur; MT: medial tibia; LT: lateral tibia. Figure S2: (a) combined cartilage radiomics model on feature select, 19 features which correspond to the optimal validation AUC were selected; (b) radiomics score plot on test data set. Figure S3: (a) combined subchondral radiomics model on feature selection, 13 features which correspond to the optimal validation AUC were selected; (b) radiomics score plot on test data set. Table S1: features and weights of the combined cartilage radiomics model. Table S2: features and their corresponding weights in the combined subchondral radiomics model. [file 4351499.f1.docx]

**Supplemental Figures**


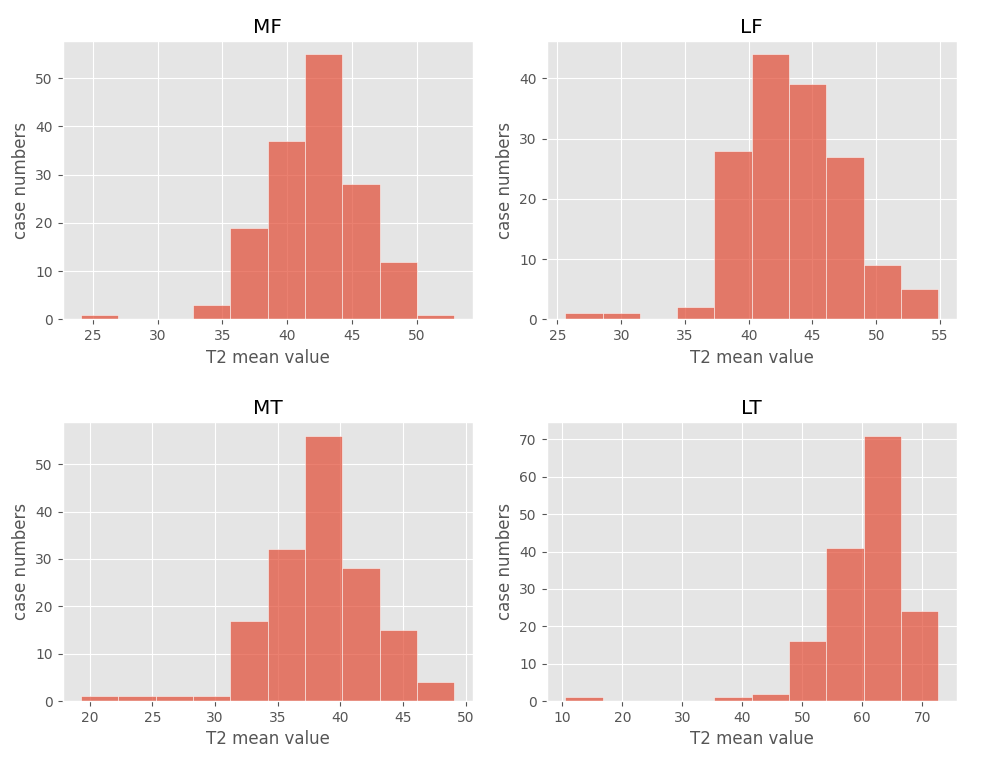


Figure S1. The distribution of cartilage T2 values of different compartments of the knee. MF, medial femur; LF, lateral femur; MT, medial tibia; LT, lateral tibia.


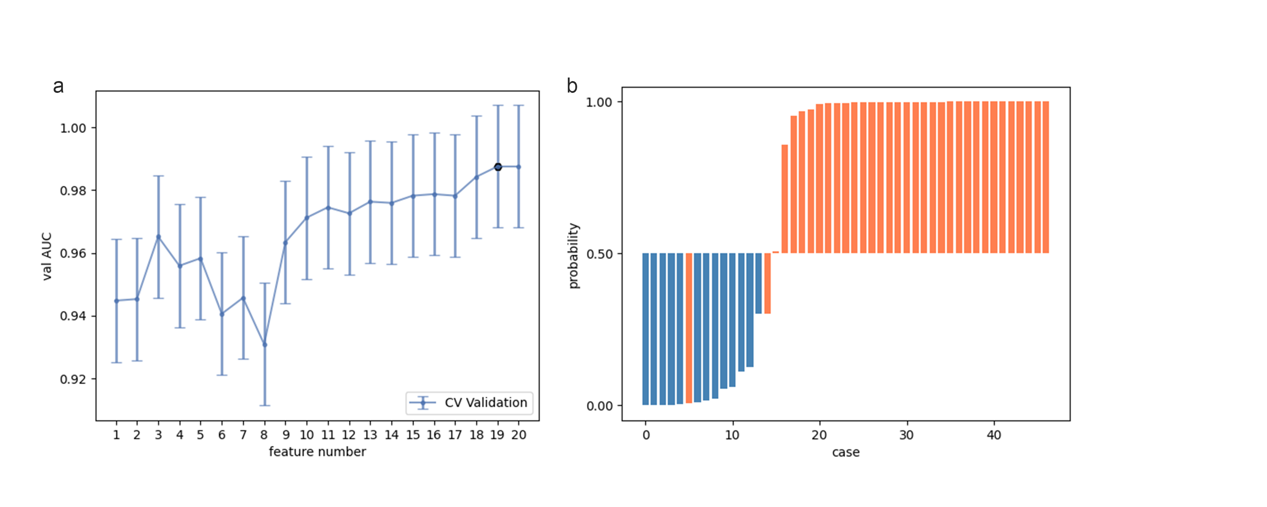


Figure S2. a. combined cartilage radiomics model on feature select, 19 features which correspond to the optimal validation AUC were selected; b. radiomics score plot on test data set.


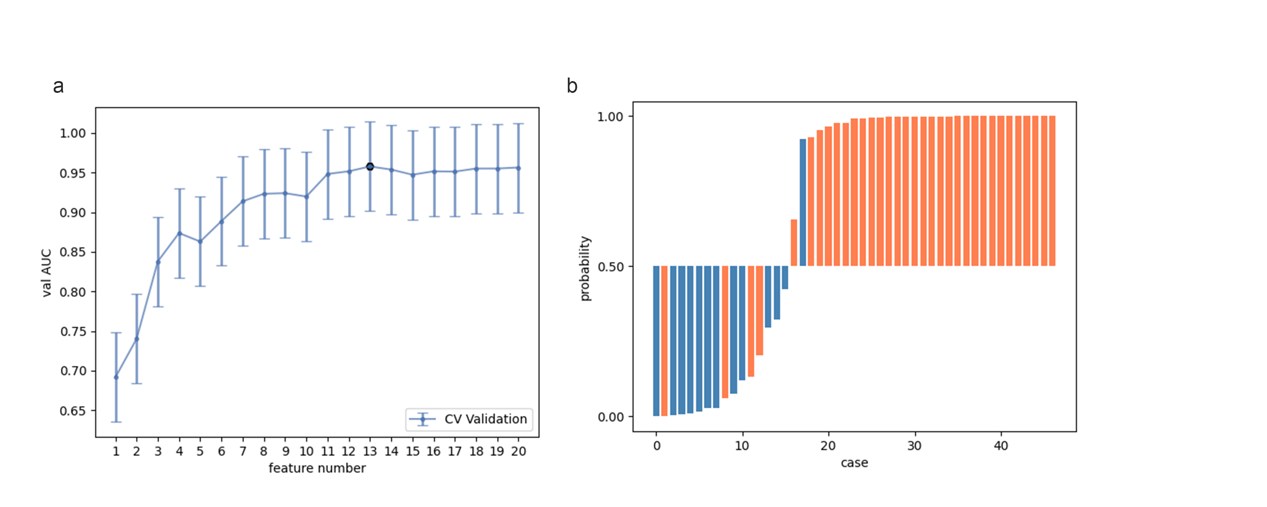


Figure S3. a. Combined subchondral radiomics model on feature selection, 13 features which correspond to the optimal validation AUC were selected; b. radiomics score plot on test data set.

**Supplemental Tables**

Table S1: Features and weights of the combined cartilage radiomics model

| Feature name | Coef in model |
| --- | --- |
| MT_log-sigma-1-0-mm-3D_glrlm_GrayLevelNonUniformity | -4.400 |
| MT_original_glszm_SmallAreaLowGrayLevelEmphasi | 2.584 |
| LT_log-sigma-3-0-mm-3D_glcm_ClusterShade | -3.656 |
| LT_wavelet-HHL_firstorder_Minimum | -4.967 |
| LT_wavelet-HHL_glcm_DifferenceAverage | 4.5696 |
| MF_log-sigma-1-0-mm-3D_glcm_DifferenceAverage | 5.3305 |
| MF_log-sigma-1-0-mm-3D_glcm_SumAverage | 6.9995 |
| MF_log-sigma-1-0-mm-3D_gldm_DependenceNonUniformityNormalized | 2.9376 |
| MF_log-sigma-3-0-mm-3D_glszm_LargeAreaEmphasis | -2.255 |
| MF_log-sigma-5-0-mm-3D_glcm_DifferenceAverage | 3.3685 |
| LF_original_glrlm_LongRunLowGrayLevelEmphasis | -2.382 |
| LF_original_glszm_SmallAreaEmphasis | -5.088 |
| LF_wavelet-HHL_glcm_SumSquares | 2.1103 |
| LF_wavelet-HHL_glszm_SmallAreaHighGrayLevelEmphasis | -7.328 |
| LF_wavelet-HLH_firstorder_90Percentile | -3.779 |
| LF_wavelet-HLH_firstorder_Entropy | -2.715 |
| LF_wavelet-HLH_firstorder_Median | -3.774 |
| LF_wavelet-HLH_glcm_Idn | -3.872 |
| LF_wavelet-HLH_gldm_DependenceNonUniformityNormalized | -1.352 |

Glrlm, Gray Level RunLength Matrix; glszm, Gray Level SizeZone Matrix; glcm, Gray LevelCo-occurrence Matrix; gldm, Gray LevelDependence Matrix

Table S2: Features and their corresponding weights in the combined subchondral radiomics model

| Feature name | Coef. in model |
| --- | --- |
| MT_log-sigma-1-0-mm-3D_glszm_ZoneEntropy | 14.751 |
| LF_original_firstorder_Energy | 14.732 |
| MT_log-sigma-1-0-mm-3D_gldm_SmallDependenceEmphasis | -3.679 |
| LT_log-sigma-5-0-mm-3D_firstorder_Mean | 6.879 |
| MF_log-sigma-5-0-mm-3D_firstorder_InterquartileRange | -0.258 |
| LF_log-sigma-3-0-mm-3D_glrlm_RunLengthNonUniformityNormalized | -10.127 |
| LF_log-sigma-3-0-mm-3D_firstorder_90Percentile | -4.363 |
| LF_log-sigma-3-0-mm-3D_firstorder_90Percentile | 6.313 |
| MF_log-sigma-3-0-mm-3D_glrlm_ShortRunLowGrayLevelEmphasis | 3.425 |
| LT_log-sigma-1-0-mm-3D_firstorder_Kurtosis | 5.810 |
| LT_log-sigma-1-0-mm-3D_firstorder_Entropy | 0.418 |
| MF_log-sigma-3-0-mm-3D_glszm_GrayLevelNonUniformity | 4.174 |
| LF_log-sigma-1-0-mm-3D_glcm_MCC | 9.211 |

Glrlm, Gray Level RunLength Matrix; glszm, Gray Level SizeZone Matrix; glcm, Gray LevelCo-occurrence Matrix; gldm, Gray LevelDependence Matrix
